# An incidental windsock diverticulum

**DOI:** 10.1002/ccr3.1458

**Published:** 2018-03-08

**Authors:** Vaishnavi Boppana, Abimbola Adike, Uche Adike, Rahul Pannala

**Affiliations:** ^1^ Department of Gastroenterology and Hepatology Mayo Clinic Arizona Scottsdale Arizona; ^2^ Scottsdale Community College Scottsdale Arizona

**Keywords:** Diverticulum, duodenum, intraluminal, windsock

## Abstract

Windsock diverticulum is a rare anomalous finding of a true intraluminal duodenal diverticulum. While complications of bowel obstruction, bleeding, and pancreatitis may occur, most patients are asymptomatic. Surgical or endoscopic management may be pursued when complications develop.

## Case Description

A 55‐year‐old gentleman with a long‐standing history of gastroesophageal reflux disease presented with new onset of dysphagia to solids. On esophagogastroduodenoscopy, there was no cause found for dysphagia but he was incidentally found to have a prominent infrapapillary fold in the second part of duodenum. This was then re‐examined at a later date with a side‐viewing scope, revealing a 3‐ to 4‐cm diverticulum in the second portion of the duodenum.

## Discussion

Windsock, also called “finger‐of‐glove,” is a true intraluminal diverticulum. It is formed due to incomplete recanalization of embryologic foregut leaving a fenestrated membrane within the lumen of *duodenum* 1.

In most cases, as in this case, it is asymptomatic and requires no intervention 1. Complications are rare and may include partial bowel obstruction, gastrointestinal bleeding, and pancreatitis 2, 3. When complications develop, endoscopic management may be attempted with a diverticulectomy using a polypectomy snare and then incising the duodenal septum using a needle knife 1.

## Authorship

Drs. Boppana and Adike wrote and edited the written content. Uche Adike edited the video content. Dr. Pannala was the performing endoscopist who identified the diverticulum, provided overall guidance, and approved the final version of the manuscript.

## Conflict of Interests

None declared.

## Supporting information


**Video S1.** A short video of an incidental duodenal diverticulum.
